# Insufficient hypothalamic angiotensin-converting enzyme 2 is associated with hypertension in SHR rats

**DOI:** 10.18632/oncotarget.15666

**Published:** 2017-02-24

**Authors:** Kun Wang, Yuanyuan Xu, Weiwei Yang, Yuanshu Zhang

**Affiliations:** ^1^ College of Veterinary Medicine, Nanjing Agricultural University, Nanjing 210095, China

**Keywords:** SHR, ACE2, hypothalamus, pituitary, hypertension

## Abstract

Angiotensin-Converting Enzyme 2 (ACE2) is a key enzyme in the renin-angiotensin system (RAS), which is implicated in the pathogenesis of hypertension and other cardiovascular diseases. In this study we investigated the expression of ACE2 in the hypothalamus and pituitary tissues and its relationship to hypertension by comparing them in male WKY and SHR rats. We observed that the plasma levels of corticotrophin releasing hormone (CRH), adrenocorticotropic hormone (ACTH) and aldosterone (ALD) were all lower in SHR than WKY rats (P<0.05), whereas plasma angiotensin II (AngII) levels were higher in SHR rats (P<0.05). Levels of ACE mRNA and protein were higher in the hypothalamus of SHR than WKY rats (P<0.05). By contrast, hypothalamic expression of ACE2 protein was lower in SHR rats (P<0.05), despite comparable mRNA levels in SHR and WKY rats. There were no differences in the expression levels of ACE, ACE2, AT1 or Mas mRNA in the pituitaries of SHR and WKY rats (P>0.05). These results suggest that insufficiency of hypothalamic ACE2 is associated with hypertension in SHR rats.

## INTRODUCTION

The role of the renin–angiotensin system (RAS) in the pathogenesis of hypertension and other cardiovascular diseases is widely acknowledged [1]. However, the traditional view that Ang II is the sole key effector peptide of the RAS has been questioned by the subsequent discovery of angiotensin-converting enzyme 2 (ACE2) [2, 3] and the growing evidence for a physiological role for Angiotensin-(1–7) [4]. Angiotensin-converting enzyme 2 (ACE2) is a homologue of the angiotensin-converting enzyme (ACE) that catalyzes Ang II into Ang-(1–7). Angiotensin-(1–7) is a peptide that binds to the G-protein-coupled receptor Mas (MasR) and initiates vasodilator and anti-proliferative responses [5]. Also, Ang-(1–7) antagonizes the cardiovascular actions of Ang II [6].

RAS is associated with hypertension in the spontaneously hypertensive rats (SHR) [7]. Cardiac ACE2 was suppressed and ACE upregulated in the SHR compared to WKY rats [8]. Moreover, it was implicated in pro-inflammatory cytokines (PICs) such as tumor necrosis factor (TNF)-a, interleukin (IL)-6, and IL-1b both centrally and in the periphery [9, 10].

The RAS system is expressed in many tissues such as pancreas, heart, liver, lungs, adipose tissue and brain and regulates blood pressure and fibrosis in these tissues [11, 12]. There is evidence that the brain RAS has a critical role in regulating blood pressure, water balance and endocrine secretion. Recent studies have demonstrated ACE2 mRNA in the rat medulla oblongata and detected ACE2 activity in the mouse brain [13, 14]. Lin and co-workers used *in situ* hybridization to measure ACE2 mRNA in the brainstem and demonstrated a relationship between the Angiotensin type 1a receptor (AT1a) and ACE2 expression [15]. ACE2 has also been implicated in attenuating hypertension in the brain and the hypothalamus [16, 17]. However, the role of ACE2 in the WKY hypothalamic-pituitary axis has not been investigated. Since the levels of angiotensin (1-7) were higher in the hypophyseal portal plasma compared to matched jugular plasma samples, a potential role for ACE2 within the hypothalamic-pituitary axis was postulated. Therefore, we analyzed the ACE2 mRNA and protein levels in the WKY and SHR rat HPA along with the plasma levels of CRH, ACTH, ALD and AngII to understand the link between ACE2 expression in the HPA and hypertension.

## RESULTS

### Systolic blood pressure, diastolic blood pressure, heart rate, and body weight

**Figure 1:**
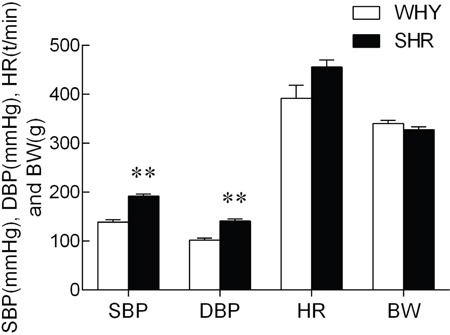
SBP, SDP, HR and BW of SHR compared with WKY Note: compared with WKY group, ** P<0.01.

Systolic blood pressure, Diastolic blood pressure, Heart rate, and Body weight were shown in Figure 1. Compared with WKY, the systolic blood pressure (SBP) and diastolic blood pressure (SDP) of SHR were increased significantly. However, the resting heart rate and body weights did not differ between two groups.

### Plasma CRH, ACTH, ALD and Ang II levels

**Figure 2:**
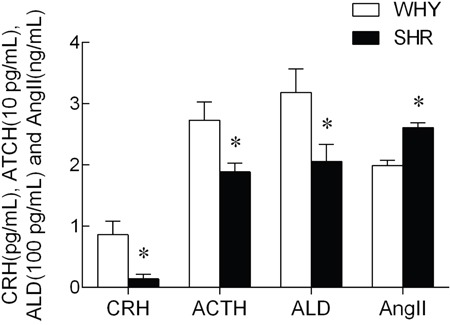
The content of CRH, ACTH, ALD and AngII in plasma from SHR compared with WKY Note: compared with WKY, * P<0.05.

Levels of CRH, ACTH, ALD and AngII in plasma were shown in Figure 2. Unpaired t-test indicated that plasma CRH, ACTH, and ALD levels decreased significantly in SHR group, In contrast, Ang II level in plasma increased markedly.

### RAS gene expression in hypothalamus and pituitary

**Figure 3:**
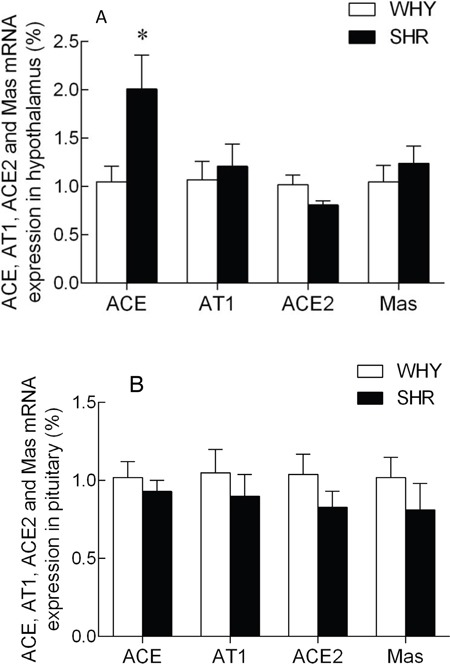
The expression of ACE, AT1R, ACE2 and MasR mRNA in hypothalamus and pituitary of SHR compared with WKY Note: A: the expression of ACE, AT1_A_, ACE2 and MasR mRNA in SHR hypothalamus compared with that of WKY group, * P<0.05; B: the expression of ACE, AT1_B_, ACE2 and MasR mRNA in SHR pituitary compared with that of WKY group, * P<0.05.

The expression of ACE, AT1R, ACE2 and MasR mRNA levels in the hypothalamus were illustrated in Figure 3A. Compared with the WKY group, the ACE mRNA levels of SHR were increased significantly, while the ACE2 mRNA levels showed a non-significant change between two groups. The AT1R and MasR mRNA were no observably changed. The expression of ACE, AT1R, ACE2 and MasR mRNA levels in the pituitary are illustrated in Figure 3B. Compared with the WKY group, the ACE, AT1R, ACE2, and MasR mRNA levels of SHR were not significantly changed.

**Figure 4:**
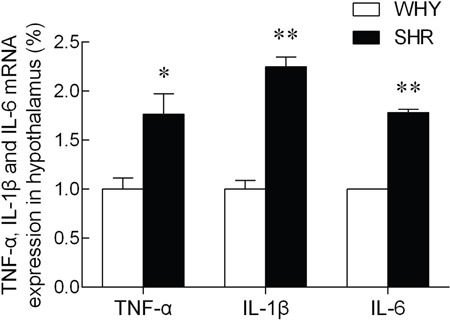
The expression of TNF-α, IL-1β and IL-6 mRNA in hypothalamus Note: compared with WKY group, * P<0.05, ** P<0.01.

The expression of TNF-α, IL-1β and IL-6 mRNA levels in the hypothalamus were illustrated in Figure 4. Compared with the WKY group, the TNF-α, IL-1β and IL-6 mRNA levels of SHR were increased significantly.

### ACE2 and ACE protein expression in hypothalamus

**Figure 5:**
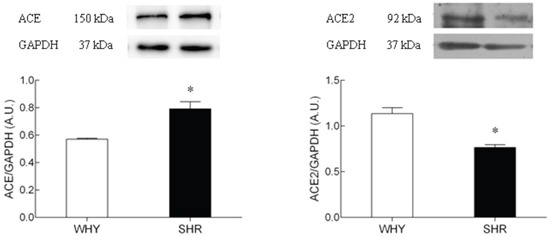
The protein expression of ACE2 on hypothalamus of SHR compared with WKY Lane 1: WKY group; Lane2: SHR group. The sample proteins have been run under the same experimental conditions, * P<0.05.

To evaluate whether the decrease of ACE and ACE2 mRNA was associated with changes in protein, western blot was performed (Figure 5). Although the ACE2 mRNA in hypothalamus between two groups was not significantly altered, the ACE2 protein expression in SHR was significantly inhibited compared with that of WKY. The ACE protein expression in SHR was significantly higher than that of WKY. The sample proteins have been run under the same experimental conditions.

## DISCUSSION

The RAS is an attractive therapeutic target for management of cardiovascular disease [18]. Renin and ACE activity are used as biomarkers for disease states such as hypertension, diabetes, heart failure and renal diseases [19, 20, 21, 22]. However, recent data indicates that ACE2 has an opposing effect to that of ACE which could have novel implications regarding the role of RAS in hypertension and cardiovascular diseases [23, 24, 25]. Our data showed that ACE2 was expressed in both hypothalamus and pituitary of male WKY and SHR rats. The expression of RAS components has been documented in many tissues, such as heart, kidneys, pancreas, lung, adipose and brain [26, 27]. Previously, the ACE2 mRNA and protein were found to be expressed in forebrain, brainstem, subcortical organ (SFO), and ventrolateral medulla (VLM) of rat [28]. Also, ACE2 was expressed predominantly in the glial cells [4]. Although ACE2 expression has been reported in DOCA-salt hypertension mice [17, 29], its expression in the hypothalamus and pituitary of SHR is unknown. Therefore, our goal was to study the expression of ACE2 in the hypothalamus-pituitary axis of rat and its relationship with hypertension.

Our study demonstrated that ACE, AT1R, ACE2, MasR mRNA were expressed in the hypothalamus and pituitary of both, the WKY and SHR rats. The upregulation of ACE mRNA and the downregulation of ACE2 mRNA and protein in hypothalamus suggested that the balance between hypothalamic ACE2 and ACE determined blood pressure. The RAS in the nervous system is critical for regulating hypertension and cardiovascular diseases. The expression of ACE2 is suppressed in RVLM of SHR compared to WKY, whereas overexpression of ACE2 decreased the high blood pressure in SHR [30]. In addition, ACE2 overexpression in the PVN demonstrated beneficial effects against the Ang II-induced hypertensive response [10]. However, similar expression of ACE, AT1R, ACE2, MasR mRNA in the pituitary of both groups of mice indicated that the pituitary RAS was not involved in the hypertension demonstrated by SHR rats.

The higher plasma levels of AngII in the SHR compared to WKY showed that the ACE-AngII-AT1R axis was hyperactive in the hypothalamus of SHR where theACE2 protein was insufficient. ACE is a key factor of RAS and generates AngII that promotes the secretion of vasopressin that causes vasoconstriction [31]. In addition, Ang (1–7), a product of ACE2, which inhibits AngII, was highly expressed in medulla oblongata and hypothalamus [32]. Elevated AngII levels induced inflammation [33], whereas overexpression of ACE2 reduced local inflammation in the hypothalamus [10]. In this context, we observed that the mRNA levels of TNF-α, IL-1β and IL-6 were enhanced in SHR compared to WKY in the hypothalamus.

ACTH increases blood pressure by inducing the release of glucocorticoids and probably aldosterone from the adrenal cortex into circulation [34]. In this study, we observed that the levels of CRH, ACTH, and ALD in the plasma were lower in SHR rats compared to WKY (P<0.05), suggesting that the activity of the HPA axis was reduced in chronic hypertension.

In summary, our data suggested that the ACE-AngII-AT1R axis was hyperactive in the hypothalamus RAS of SHR where the ACE2 protein was relatively insufficient and the pituitary was not involved in the hypertension of SHR rats.

## MATERIALS AND METHODS

### Animals

All animal experiments were performed in accordance with relevant guidelines and regulations of the Institutional Ethical Committee of Nanjing Agricultural University Institutional Animal Care and Use. All animal experimental protocols were approved by Institutional Ethical Committee of Nanjing Agricultural University. Fourteen week old male SHR (n=8) and WKY rats(n=8) from Shanghai Experimental Animal Center (Shanghai, China) were housed in individual cages with free access to water and rat chow ( Xie-tong Medicine Biological Engineering Co., Ltd., Jiangsu, China) for one week. Then all rats were euthanized by cervical dislocation and trunk blood samples collected. Hypothalamus and pituitaries were collected and frozen at -80°C.

### Blood pressure, heart rate, and body weights

The rat systolic blood pressure (SBP), diastolic blood pressure (SDP) and heart rate (HR) was measured using a multi-channel noninvasive pressure system under a conscious resting state. The body weight was measured with a weighing-machine.

### Plasma radioimmunoassay

Plasma CRH, ACTH, ALD, and Ang II levels were determined with commercially available radioimmunoassay kits of CRH, ACTH, ALD and Ang II (Beifang BioTech Co. Ltd, Beijing, China) according to the manufacturer's protocol. The radioactive precipitation was counted by a γ-counter. The intra- and inter-assay coefficients of variation were less than 10% and 15%.

### Real-time quantitative PCR assay

RNA was extracted from the hypothalamus and pituitary using an RNeasy mini kit (Tiangen). The cDNA was generated by using PrimeScript RT reagent Kit (TaKaRa) with oligo-dT and random primer. The real-time PCR reaction was then performed in an ABI Prism 7700 Detection System (ABI, USA). The sequences of rat ACE, ACE2, AT1R, MasR, TNF-α, IL-1β, IL-6 and β-actin primers were listed in Table 1. Message levels were normalized to β-actin levels in each experiment.

**Table 1:** Nucleotide Sequences of PCR Primers

Gene	GeneBank Accession Number	Orientation	Primers Sequences (5′-3′)	Product Size (bp)
ACE2	AY881244	Forward	5′-AATCGTAGGCTCTGGGCTTGG-3′	182
		Reverse	5′-TTCGA TCAACTGGTTTCGGTTGTA-3′	
Mas	NM012757	Forward	5′-TGACAGCCATCAGTGTGGAGA-3′	116
		Reverse	5′-GCATGAAAGTGCCCACAGGA-3′	
ACE	NM012544	Forward	5′-ATGCCTCTGCGTGGGACTTC-3′	112
		Reverse	5′-TACTGCACGTGGCCCATCTC-3′	
AT1R	NM030985	Forward	5′-CCCACTCAAGCCTGTCTACGAA-3′	120
		Reverse	5′-GTGTGCTTTGAACCTGTCACTCC-3′	
TNF-α	NM013693	Forward	5′-GAGTGACAAGCCTGTAGCCC-3′	253
		Reverse	5′-GAGGTTGACTTTCTCCTGGTAT-3′	
IL-1β	NM031512	Forward	5′-GTGCGTGCTTAGATGTTGCT-3′	265
		Reverse	5′-TGTTCCTACTTACCTTGTTGGC-3′	
IL-6	NM031168	Forward	5′-ATGGCAATTCTGATTGTATG-3′	212
		Reverse	5′-GACTCTGGCTTTGTCTTTCT-3′	
β-actin	AF122902	Forward	5′-CCCTGTGCTGCTCACCGA-3′	198
		Reverse	5′-ACAGTGTGGGTGACCCCGTC-3′	

### Western blot analysis

Tissue was homogenized in RIPA lysis buffer containing protease inhibitor mixture [18]. Protein concentration was determined with BCA Protein Assay Kit (Beyotime Institute of Biotechnology, Shanghai, China). Normalized amount of sample proteins (60μg/lane) were separated by 8% SDS-PAGE gel and transferred to PVDF membrane with a Pegasus semi-dry-blotter (Bio-rad, USA). After blocking in 5% skim milk in TBS with 0.1% tween-20 (TBST) at room temperature, the membrane was incubated with diluted primary antibody against ACE2 (1:400, Santa Cruz Biotechnology Inc, USA) or ACE (1:1000, Abcam, USA) over night at 4°C. After being washed four times with TBST, the membrane was incubated with HRP-conjugated anti-rabbit IgG (1:10000, Boster Biological Technology, Ltd, Wuhan) for 1 h at room temperature. After being washed with TBST, specific immunoreactive proteins were detected by enhanced ECL substrate (Beyotime Institute of Biotechnology, Shanghai, China) on a chemiluminescence system (Tanon, Shanghai, China).

### Statistical analysis

Data were analyzed by t-test using SPSS version 17.0. Each value was expressed as mean ± standard error (SE). Statistical significance was accepted for P < 0.05.
